# Breast cancer heterogeneity and its implication in personalized precision therapy

**DOI:** 10.1186/s40164-022-00363-1

**Published:** 2023-01-09

**Authors:** Liantao Guo, Deguang Kong, Jianhua Liu, Ling Zhan, Lan Luo, Weijie Zheng, Qingyuan Zheng, Chuang Chen, Shengrong Sun

**Affiliations:** 1 Department of Breast and Thyroid Surgery, Renmin Hospital of Wuhan University, No. 238 Jiefang Road, Wuchang District, Wuhan, 430060 Hubei China; 2 Department of Urology, Renmin Hospital of Wuhan University, No. 238 Jiefang Road, Wuchang District, Wuhan, 430060 Hubei China; 3 Department of Breast Surgery, The Affiliated Hospital of Guizhou Medical University, No. 28 Guiyi Road, Yunyan District, Guiyang, 550001 Guizhou China

**Keywords:** Breast cancer, Heterogeneity, Cell interaction, Cell competition, Precision therapy

## Abstract

Breast cancer heterogeneity determines cancer progression, treatment effects, and prognosis. However, the precise mechanism for this heterogeneity remains unknown owing to its complexity. Here, we summarize the origins of breast cancer heterogeneity and its influence on disease progression, recurrence, and therapeutic resistance. We review the possible mechanisms of heterogeneity and the research methods used to analyze it. We also highlight the importance of cell interactions for the origins of breast cancer heterogeneity, which can be further categorized into cooperative and competitive interactions. Finally, we provide new insights into precise individual treatments based on heterogeneity.

## Introduction

**Fig. 1:**
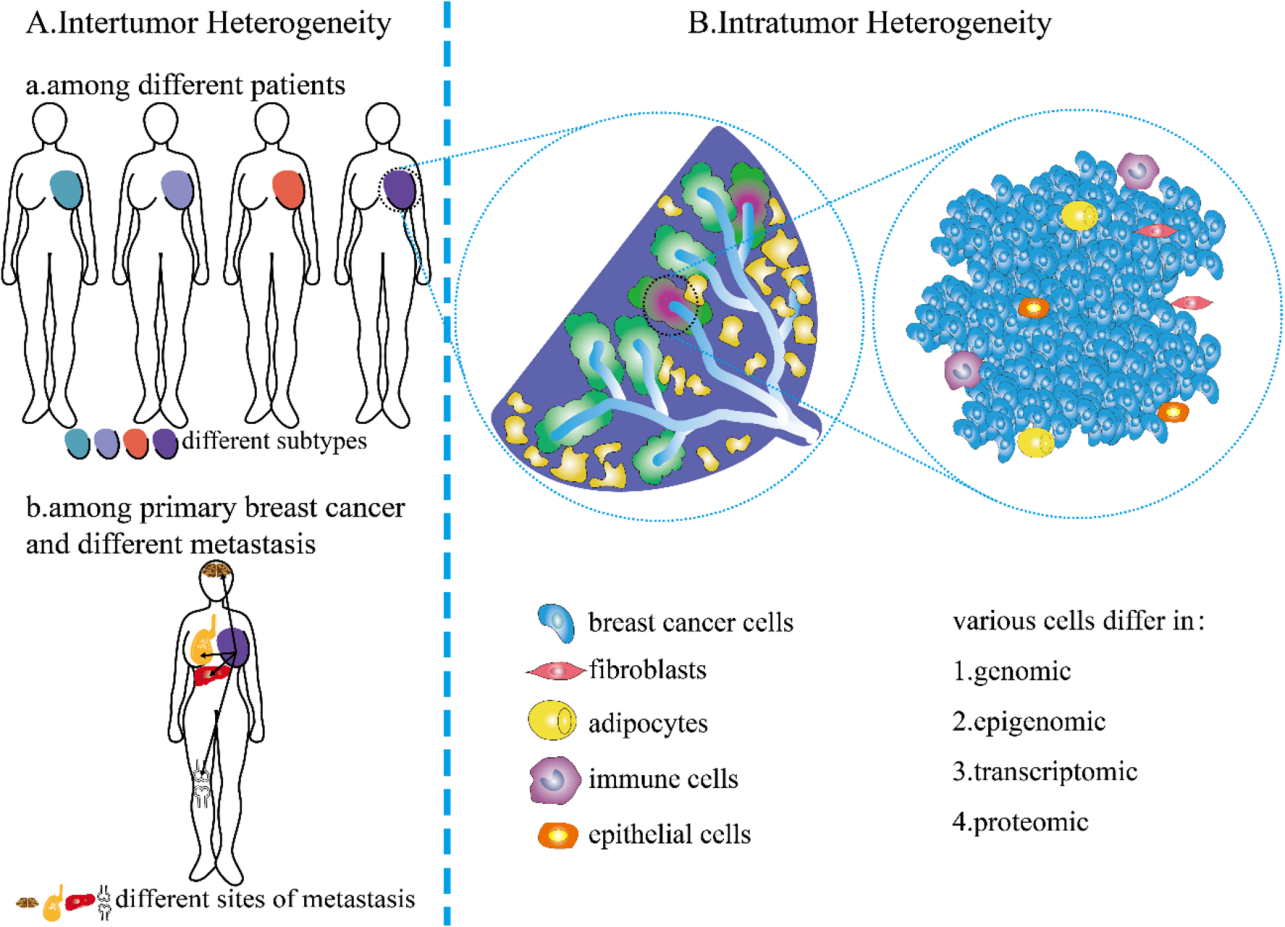
Inter- and intratumor heterogeneity of breast cancer. **A** Intertumoral heterogeneity: **a** Variation in different breast cancer subtypes among different patients; **b** Variation in different breast cancer subtypes in patients with primary breast cancer and its metastasis in the same patient. **B** Intratumor heterogeneity: variation in different cell types within a tumor

Breast cancer is the most common cancer among women worldwide, with increasing incidence. Owing to its heterogeneity [1], it is also the second leading cause of cancer-related deaths in women [2, 3]. The heterogeneity of breast cancer is attributed to differences in the genomic, epigenomic, transcriptomic, and proteomic characteristics of the cancer cells. These factors affect tumor properties such as proliferation, apoptosis, metastasis, and therapeutic response. This heterogeneity is also observed in tumor tissues among different patients or different metastases (intertumor heterogeneity) and within the individual tumor from the same patient (intratumor heterogeneity) (Fig. 1) [4]. The heterogeneity is also spatiotemporal. Temporal heterogeneity refers to the dynamic variations in the molecular makeup of cancer cells during tumor progression [5]. Spatial heterogeneity describes the distribution and interactions of different cell populations in complex tumor structures. Several scholars believe breast cancer is the most heterogeneous cancer type in women worldwide [6]. Breast cancer heterogeneity increases the difficulty of early diagnosis, treatment selection, and prognosis prediction. For instance, in situ recurrence, distant metastasis, and many clinical problems in breast cancer treatment arise from the heterogeneity [1, 6–8]. In this review, we summarize the different levels of heterogeneity, their influence on breast cancer progression, the underlying mechanisms, new research methods to measure breast cancer heterogeneity, and treatment strategies in terms of heterogeneity.

## Different levels of heterogeneity

The most fundamental manifestations and causes of heterogeneity of breast cancer are genomic, epigenomic, transcriptomic, and proteomic alterations expressed in spatial and temporal dimensions. At the genomic level, the heterogeneity comprises of mutations [9] and copy number aberrations (CNAs) [10]. At the epigenomic level, the heterogeneity comprises of transposase-accessible chromatin [10], DNase [11], nucleosome occupancy [12], methylome [12], and so on. At the transcriptomic level, the heterogeneity refers to the stochastic gene expression [9]. At the proteomic level, the heterogeneity comprises of protein modifications and signal transduction [9]. The Cancer Genome Atlas [13], Molecular Taxonomy of Breast Cancer International Consortium [14], and more recently, single-cell profiling [15–19] have revealed intertumor and intratumor heterogeneity also includes temporal and spatial variations. Changes in the genome, epigenome, transcriptome, and proteome heterogeneity can be reflected in various aspects, such as biomarkers, metabolism, cell cycle, tumor microenvironment (TME), epithelial–mesenchymal transition (EMT), circulating tumor cells (CTCs), and clinical pathology.

### Biomarkers

Regarding biomarker heterogeneity, the American Society of Clinical Oncology/College of American Pathologists recommends detection of the expression levels of estrogen receptor (ER), progesterone receptor (PR), and human epidermal growth factor receptor 2 (HER2) in all invasive breast carcinomas by immunohistochemistry [20, 21]. ER and PR are often referred to collectively as hormone receptors (HRs). ER is produced by three genes, named *ERα*, *ERβ*, and membrane receptor G protein-coupled receptor 30 (*GPR30*) [22]. PR is produced by two genes, *PRA* and *PRB* [23]. HER2 is produced by *ERBB2* [24]. Based on these biomarkers, breast cancers are typically classified into four subtypes: luminal A (ER^+^, PR^+^, and HER2^–^), luminal B (ER^+^, PR^+^, and HER2^+^), HER2-enriched (ER^–^, PR^–^, and HER2^+^), and triple-negative breast cancers (TNBC) (ER^–^, PR^–^, and HER2^–^) [25–28]. According to Fudan University Shanghai Cancer Center classification criteria, TNBC can further be categorized into immunomodulatory (IM), luminal androgen receptor (LAR), mesenchymal-like (MES), basal-like and immune-suppressed (BLIS) subtypes [29]. Recently, the claudin-low subtype has been defined as mesenchymal cell-like, stem cell-like, and has high expression of immune response genes [30]. It was demonstrated that the claudin-low subtype is an additional complex phenotype that may permeate breast tumors of different subtypes [31]. Furthermore, HR^+^/HER2^+^ tumors behave differently, with some resembling more luminal A subtype and others resembling HER2-enriched subtype [32]. Some scholars believe that it should be considered an individual subtype. Breast cancer treatment is often based on the ER, PR, and HER2 status in primary cancer. However, increasing data have shown substantial discordance between the primary sites or the receptor profiles of primary breast cancers and their distant metastases [33–41]. This discordance can also be observed between different metastatic sites [42]. This conversion may reflect sampling or treatment effects; however, they may also indicate an alteration in cancer biology and heterogeneity.

### Metabolism

Recent studies have demonstrated that metabolic levels differ among cells in a tumor mass, resulting from differences in various metabolic pathways [43]. The degree of biomarker positivity is negatively correlated with the intensity of metabolism and aggressive clinical behavior [44, 45]. For example, TNBC, with a more aggressive clinical course, showed increased demand for nicotinamide, 1-ribosyl-nicotinamide, and NAD^+^ than other types of breast cancer. Compared with primary cancer cells, metastatic cells shift their metabolic levels with lower glucose metabolism [46]. In contrast, metastatic cells have higher levels of fumarate, malate, and succinyl-carnitine [46]. Furthermore, metastatic cells have higher fatty acid and amino acid metabolism than primary cancer cells [46].

### Cell cycle

An uncontrolled cell cycle is a hallmark of breast cancer [47]. Nevertheless, the cell cycle progression within breast cancer cells varies considerably [48]. Using cell cycle phase analysis, researchers have found that most cancer cells in tumors do not undergo the conventional cell cycle phases [48] and are resistant to treatment. Compared with other subtypes of breast cancer, TNBC show a higher proportion of mitoses in the overall cycling cells [49]. Furthermore, tumor size was demonstrated to positively affect the proportion of proliferated cells.

### TME

The TME plays a significant role in tumor growth and metastasis and has always been an important obstacle during tumor therapy. The TME has many heterogeneous cell populations, including fibroblasts, adipocytes, immune cells, epithelial cells, pericytes, and extracellular matrix components [50]. Interactions between tumor cells and their microenvironment contribute to tumor heterogeneity, thus promoting tumor progression [51].

### EMT, CSCs, and CTCs

Generally, EMT is regarded as the first and foremost step in the cancer metastasis [52, 53]. It is considered a heterogeneous phenomenon [54] that can also be involved in embryonic development and wound healing. EMT correlates with cancer stem cell (CSC) plasticity and therapeutic resistance [55, 56]. Increasing evidence indicates that EMT promotes breast cancer metastasis [57]. CTCs represent the intermediate stage of metastasis [58]. CTCs are found in the peripheral blood of breast cancer patients with metastatic lesions [58]. They can be derived from primary and metastatic sites [58]. CTCs exhibit high inter- and intra-patient heterogeneity compared with normal breast cancer cells [59]. Therefore, the analysis of CTCs can be an essential tool for studying the heterogeneity between cells from the primary and metastatic sites. Because CTCs can be obtained by minimally invasive peripheral blood extraction, they can be collected continuously to monitor molecular changes as the disease progresses and, significantly, under therapeutic pressure [60]. Thus, analysis of a single CTC enhances the assessment of heterogeneity.

### Microcirculation

Heterogeneity in tumor microcirculation is caused by the combination or addition of vessels, angiogenic vessels, mosaic vessels, and vasogenic mimicry [61]. As such, antiangiogenic agents alone can lead to poor efficacy and resistance**.** Vasogenic mimicry (VM) is a vascular structure formed by cancer cells that can transport blood and cancer cells to efficiently obtain oxygen and nutrients independent of endothelial cells [62, 63]. The presence of VM in breast cancer is a heterogeneous phenomenon. It was demonstrated that, compared with other subtypes, VM tends to form in TNBC [64, 65]. Furthermore, VM is associated with high tumor grade, tumor progression, invasion, metastasis, and poor prognosis [63]. Tubular type and patterned matrix type are two types of VM [63]. The tubular type may be morphologically confused with vessels lined with endothelial cells. Patterned matrix type is not morphologically or topologically similar to vessels at all [61], and is characteristic of highly invasive tumors in which therapeutic regimens that target angiogenesis may be ineffective, resulting in recurrence and metastasis [61].

### Clinical pathology

In a clinical and histopathologic evaluation of heterogeneity, the American Joint Committee on Cancer incorporated a tumor, lymph node, metastasis (TNM) staging system [66]. TNM is scored based on tumor volume, the status of nearby lymph nodes, and the presence of distant metastasis to judge the heterogeneity and guide the prognosis and treatment of patients [67]. The grading of breast cancer also highlights its heterogeneity. Grading is assessed according to three morphological parameters: the proportion of carcinoma in glands and tubular structures, degree of nuclear pleomorphism, and mitosis rate in three hierarchical (low, medium, and high) systems [68].

## Breast cancer heterogeneity affects disease progression

Therapeutic resistance, recurrence, and metastasis are the most severe problems in the breast cancer treatment [69]. These problems cannot be completely separated from each other. Chemotherapy can kill sensitive cancer cells; however, the resistant cells left behind can cause tumor recurrence (or relapse) and even cancer metastasis [70].

### Therapeutic resistance

In cancer, genomic instability results in a significant degree of intercellular genetic heterogeneity. According to the tumor stem cell theory, the original genetic variation can lead to changes in epigenetic factors [70] and the cell cycle. Gene products that are directly involved in the development of drug resistance may be increased or diminished following changes in these factors [70]. Therefore, the mechanisms underlying the heterogeneity must be studied to address therapeutic resistance. Neoadjuvant chemotherapy for many patients with TNBC, is effective in approximately half of all cases [71, 72]. The genomic and molecular basis of chemotherapy resistance in patients with TNBC is still poorly understood. Kim et al. applied single-cell DNA and RNA sequencing in addition to bulk exome sequencing of samples from 20 patients with TNBC during NAC [16] and discovered a new chemoresistance model containing two modes of evolution. They found the preexistence of therapy-resistant genotypes in these patients prior to NAC. Furthermore, during treatment with NAC, a small portion of genotypes selected by NAC were subjected to transcriptional reprogramming. In this model, two modes of evolution (adaptive and acquired) were in operation, resulting in therapy resistance and recurrence. Similar findings have been observed in ER^+^ breast cancer. Marta et al. performed extensive single-cell gene expression profiling of breast cancer cell line MCF7 and its derivatives, including docetaxel-resistant cells [73]. They found untreated parental cells already contained a small portion of cells in which EMT and stemness-related genes were upregulated and cell cycle-related genes were downregulated, similar to drug-resistant cells. During chemotherapy, this population may be positively selected, leading to treatment failure. In addition, epigenetic reprogramming, aberrant co-factor activity, hypoxia, stromal factors, and inflammatory and immune components are thought to contribute to the resistance to endocrine therapies.

Radiation therapy is an adjunctive therapy for many primary cancers and one of the most commonly used methods for breast cancer treatment [74]. Despite the use of advanced radiotherapy methods, radiotherapy accuracy, and fractionation schemes, radiation therapy resistance is frequently observed [75, 76] in the form of tumor progression and recurrence. Solid tumors, such as breast and lung tumors, contain markedly heterogeneous CSC populations [77]. Radiation therapy resistance in breast tumors is highly correlated with the proportion of CSCs [78]. A higher proportion of breast CSCs (BCSCs) leads to higher radioresistance and lower biological efficiency of the tumor.

As mentioned above, immune cells as a part of the TME, its subtype, percentage, and distribution vary significantly in breast cancer masses [79]. “Immune subtypes” of breast cancer can be defined as neutrophil-enriched subtypes (NES) and macrophage-enriched subtypes (MES) [79]. MES mainly consists of C–C chemokine receptor type 2 (CCR2)-dependent macrophages that respond to immune checkpoint blockade (ICB) therapy. NES contains widespread or localized immunosuppressive neutrophils (or granulocytic myeloid-derived suppressor cells) resistant to ICB therapy and includes a small number of macrophages unaffected by *CCR2* knockout. Converting MES to NES enables an initially sensitive MES model to gain ICB resistance [79].

### Recurrence and metastasis

Owing to heterogeneity, small portions of breast cancer cells resistant to chemotherapy, radiation therapy, and immune therapy are selected and survive during treatment, as discussed above. The survival of resistant cancer cells leads to treatment failure and recurrence after treatment. BCSCs are highly correlated to the recurrence of breast cancer patients even after surgery [80]. BCSCs are highly heterogeneous, and patients with higher marker expression of 74 genes, which have since been revealed by single-cell RNA sequencing, in BCSCs were more likely to show recurrence than patients with lower marker expression [81, 82]. However, different subtypes of breast cancer exhibit various recurrence risks. TNBC has a higher recurrence rate than other types of breast cancer, partially due to a lack of effective targeting strategy, especially in the first five years [83]. Tumor-infiltrating lymphocytes (TILs) are associated with survival benefits in HER2-positive and TNBC; however, in luminal/HER2-negative tumors, high TILs are negatively associated with survival benefits. Thus, it is challenging to determine the prognosis of treatment and the recurrence risk owing to heterogeneity.

More than 90% of breast cancer-related deaths result from metastasis [13, 84, 85]. The 5-year overall survival rate of patients with breast cancer without metastasis is greater than 80% [86, 87]. Circulating breast cancer cells can be divided into various cell types based on differences in biomarker status, epithelial/mesenchymal phenotype, aggregation status, and other factors [88, 89]. RNA sequencing has revealed that weakly migratory subpopulations are largely epithelial and highly migratory subpopulations are largely mesenchymal [88]. Interestingly, the metastases of weakly migratory subpopulations are significantly more frequent than those of highly migratory subpopulations when injected in situ [88]. Hapach et al. found that weakly migratory cells gather as CTC clusters in the circulation rather than remain as a single CTC. The higher expression of E-cadherin also contributed to increased metastasis [88]. CTCs may exploit their genetic potential and communicate with the surrounding environment, such as chemokine systems, blood cells, and extracellular matrix components, to regulate organ-specific metastasis of breast cancer [89]. VM may also play an essential role in metastasis. It was found that patients with VM tended to have a higher rate of hematogenous recurrence and a lower 5-year survival rate. By inhibiting VM, primary tumor growth and lung metastasis are reduced [90].

## Cell interactions contribute to breast cancer heterogeneity

Previous research has mainly focused on the intracellular autonomic changes that lead to abnormal activity of cancer cells, which is a hallmark of cancer. However, there is increasing recognition of how non-cell-autonomous factors influence cancer cell survival, growth, and proliferation and their function and contribution to cancer. An increasing number of studies have reported competitive or cooperative interactions between cells in recent years.

In some cases, breast cancer subclones may interact to gain a selective growth advantage, and interclonal cooperation is essential for cancer maintenance [91]. Cooperation between different cancer cell subtypes may also result in a malignant phenotype [92]. In contrast, more aggressive clones and subclones can eliminate less aggressive and normal host cells through competitive interactions. Cellular competition refers to the survival of the fittest at the cellular level. The winner of the cell competition can identify and eliminate defective or damaged cells with low fitness characteristics (called "losers"), thus creating growth space and nutrients for the growth of cancer cells. Cellular competition may inhibit early carcinogenesis by eliminating mutant cells from standard wild-type cells [93–95]. Competition between cancer cells and the TME is one of the mechanisms of tumor invasion, diffusion, evolution, and formation of the intratumor [96–98] and intertumor heterogeneity [97]. This phenomenon has been linked to several cancer-related genes and thus may play an essential role in cancer. Cancer hypoxia, clonal stem cell selection, and immune cell response are thought to have a potential connection with the mechanisms that occur during cell competition [99].

**Fig. 2:**
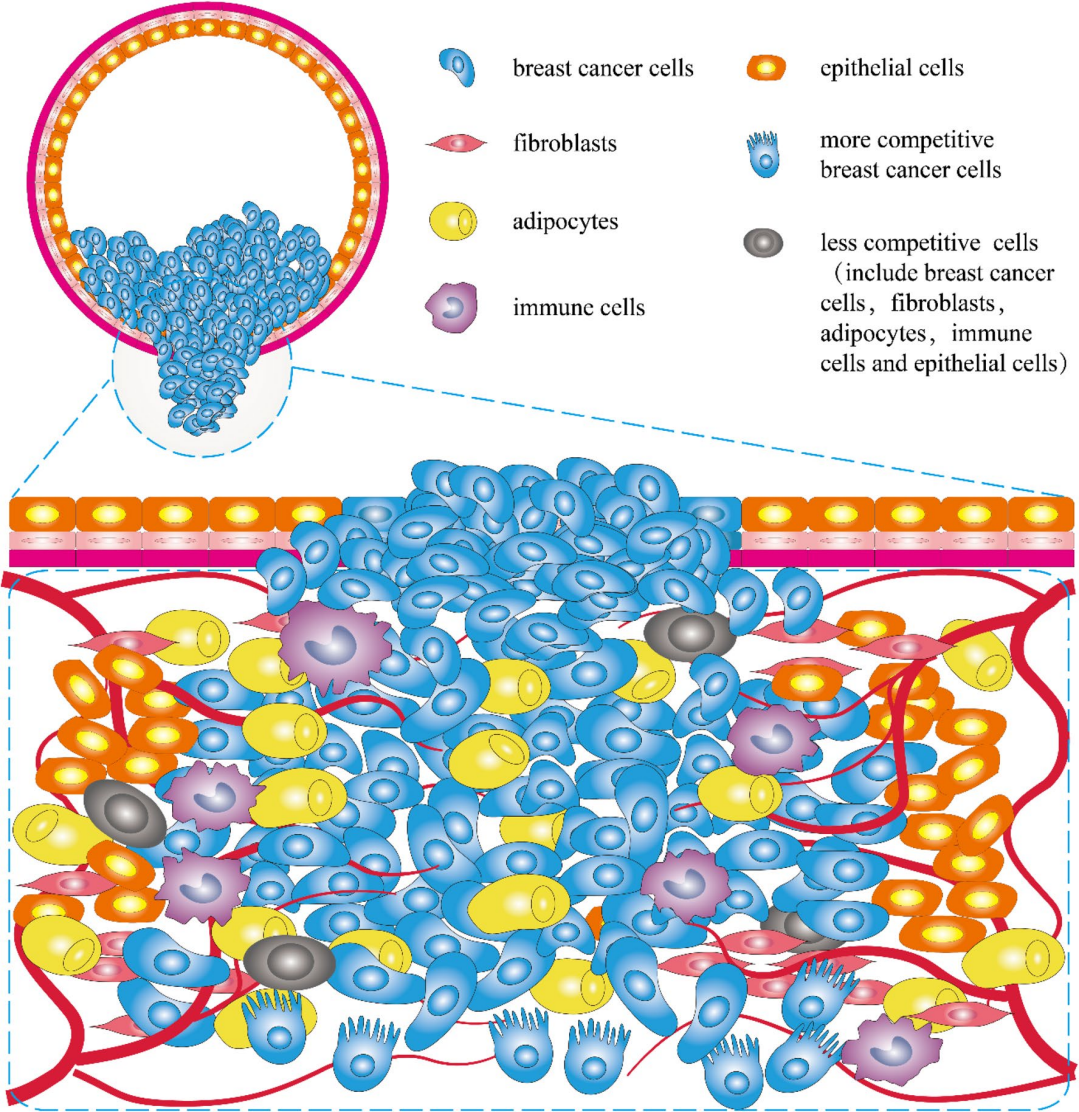
Schematic of breast cancer microenvironment. Breast cancer cells infiltrate tissues and interact with various cell types

Notably, cancer initiation and progression are not static but highly dynamic processes; therefore, subclones of cancer cells and cell interactions change over time [100]. Changing cancer cell interactions eventually produce heterogeneous cancers with various genotypes and phenotypes [101, 102] (Fig. 2). Therefore, how cell-to-cell interactions shape cancer populations in both space and time and to what extent cell interactions actively shape cancer dynamics require further exploration.

### Breast cancer cells—fibroblasts

Cancer-associated fibroblasts (CAFs) are specialized fibroblasts in the cancer microenvironment [103] derived from various cells. CAFs can stimulate cancer growth and progression by secreting stromal cell-derived factor 1 (SDF-1)/C-X-C motif ligand 12 (CXCL12) [104, 105], transforming growth factor-β (TGF-β) [105], interleukin (IL)-8 [106], and IL-32 [107]. CAFs harboring activated hypoxia-inducible factor-α (HIF-α) can promote cancer growth and lymph node metastasis via the paracrine production of recycled nutrients to feed cancer cells [108]. After coculture with CAFs, the expression levels of S100 calcium-binding protein A4 (S100A4), TGF-β, fibroblast growth factor (FGF)2, FGF7, matrix metalloproteinase (MMP)-2, MMP9, MMP11, platelet-derived growth factor (PDGF) A, PDGFB, vascular endothelial growth factor A (VEGFA), IL-6, IL-8, urokinase-type plasminogen activator (uPA), and tissue inhibitor matrix metalloproteinase 1 (TIMP1) in breast cancer cells are increased [109]. TGFβ secreted by breast cancer cells can stimulate bone morphogenetic protein (BMP) antagonist GREMLIN1 (GREM1) in CAFs [110]. GREM1 further promotes mesenchymal phenotype, stemness, and invasion by disrupting BMP/SMAD signaling in breast cancer cells [110]. CAFs can secrete exosomes containing microRNA(miR)-21, -378e, -143 [111], -16, -148a [112], and -500a-5p [113], which are transferred to breast cancer cells and subsequently promote the proliferation and metastasis of cancer cells. Interestingly, four CAF subpopulations in metastatic lymph nodes play different roles.

CAF-S1 activates the EMT of breast cancer cells through CXCL12 and TGFβ pathways, mainly playing a role in stimulating cancer cell migration. At the same time, CAF-S4 induces the invasion of breast cancer cells through NOTCH signaling in a three-dimensional space [105]. Furthermore, collagen type I secreted by CAF is related to a decrease in intratumor chemotherapeutic drug uptake, which results in resistance to chemotherapy [114, 115]. CAFs can function as a niche, allowing diffuse cancer cells to metastasize and colonize distant organs [116]. Furthermore, CAFs can remodel the extracellular matrix and immune microenvironment, inducing cancer cell resistance to immune checkpoint inhibitors (ICIs) and leading to refractory cancer [117–120]. Interestingly, breast cancer cells can induce the transformation of fibroblasts into CAFs, which in turn recruit breast cancer cells to promote cancer progression [121]. Thus, cancer cells interacting with CAFs could lead to resistance to anticancer chemotherapy and disease progression, contributing to breast cancer heterogeneity.

High-throughput live-cell imaging has revealed that normal human fibroblasts can suppress cancer cells to varying degrees [122]. The expression of phosphatase and tensin homolog (PTEN) in normal fibroblasts can suppress epithelial mammary tumors [123] through the Pten–miR-320–Ets2 axis [123, 124]. Furthermore, transplanting normal fibroblasts from a normal breast into a xenograft model of progressive proliferative breast disease can inhibit cellular invasiveness and tumor progression [125] through enhanced apoptosis. TNBC cells can provide integrin β4 (ITGB4) proteins via exosomes to induce mitophagy in the CAFs [126]. ITGB4-induced mitophagy in CAFs can promote the conversion of pyruvate to lactate to feed breast cancer cells, contributing to TNBC progression [126].

### Breast cancer cells—adipocytes

Cancer-associated adipocytes (CAAs), a main component of the TME, modify cancer cell phenotype by interacting with tumor cells [127, 128]. Moreover, leptin and adiponectin produced by adipocytes can increase the proliferation and invasion of cancer cells [129]. Adipocyte-derived IL-6 and leptin promote breast cancer metastasis by activating the Janus kinase (JAK)/signal transducer and activator of transcription 3 (STAT3) and phosphoinositide 3-kinase (PI3K)/protein kinase B (AKT) signaling pathways, thus upregulating lysyl hydroxylase (PLOD2) expression [130]. After the activation of PI3K/AKT in the breast cancer cells, the expression of IL-6, IL-1β, and tumor necrosis factor-α (TNF-α) in breast cancer cells is increased [131]. Leptin can also promote plasminogen activator inhibitor-1 expression, which is related to EMT, by activating STAT3 to promote metastasis [132]. Free fatty acids (FFAs) [133], monocyte chemoattractant protein-1 (MCP-1) [134], CC-chemokine ligand 5(CCL5) [135], and insulin-like growth factor 1 (IGF-1) [136] secreted by CAAs can induce breast cancer cells to be more invasive or more proliferative. Interestingly, higher expression of HIF-1α in adipocytes causes the loss of the ERα protein [137], which may play a role in receptor conversion and therapy resistance. In addition, a coculture study showed that adipocytes could secrete adipocyte soluble factors, which can promote higher major vault protein (MVP) expression of breast cancer cells to reduce drug accumulation in the nuclei, and efflux into the extracellular medium through extracellular vesicle secretion, thus inducing a multidrug-resistant phenotype [115]. Moreover, higher MVP expression is found at the invasive front in cancer cells closer to adipocytes, suggesting that MVP-mediated drug resistance may be clinically relevant [115]. The interactions between cancer cells and adipocytes affect cancer progression and heterogeneity.

### Breast cancer cells—immune/inflammatory cells

Inflammatory processes are associated with the initiation and progression of breast cancer, which often presents with many immune cells [138]. The infiltration of immune cells in tumors may be heterogeneous, with significant differences in immune cell types and protein composition among patients and tumor stages [139]. Tumor-associated macrophages (TAMs) are essential in TME and are involved in carcinogenesis, progression, and therapy resistance in the cancer [140–143]. IL-1β, IL-4, IL-10, IL-13, and TGF-β1, which are secreted by TAMs, are of significance in promoting tumor growth, EMT, or CSC formation [144–146]. TGF-β derived from breast cancer cells can stimulate the *uPA* gene, increase *uPA* mRNA stability, and activate uPA expression in TAMs [147]. TAMs can concentrate uPA at the cell surface by expressing urokinase receptor (uPAR), which can reshape the extracellular matrix through proteolysis, leading to the movement of breast cancer cells [148]. Breast cancer cells can downregulate the expression of epigenetic factor lysine demethylase 6B (KDM6B) in TAMs by delivering miR-138-5p via exosomes to TAMs [149]. Then, the decrease of KDM6B stimulates M2 polarization of the TAMs [149]. M2 macrophages can secrete chitinase 3-like protein 1 (CHI3L1) to interact with interleukin-13 receptor α2 (IL-13Rα2) chain on the plasma membranes of cancer cells to activate the mitogen-activated protein kinase (MAPK) signaling pathway, by which the expression of *MMP* genes are upregulated, leading to the promotion of the metastasis of breast cancer cells [150]. Breast cancer cells can downregulate the expression of protein phosphatase 2 catalytic subunit alpha (PPP2CA) in TAMs by delivering miR-183-5p via exosomes to TAMs [151]. *PPP2CA* downregulation enhances NF-κB signaling and promotes macrophage expression of IL-1β, IL-6, and TNF-α, which contributed to tumor progression [151].

In contrast, immune/inflammatory cells are recruited into tumor tissues during tumor growth to facilitate the overgrowth of cancer cells and suppress them. Activated T cells, B cells, natural killer (NK) cells, activated monocytes, dendritic cells, myeloid cells, and thymocyte subsets can express programmed cell death protein 1 (PD-1), an inhibitory immune checkpoint in breast cancer [152]. PD-L1, a PD-1 ligand, is expressed on cancer and immune cells and is associated with histological grade, hormone status, and prognosis of breast cancer [153, 154]. The expression of PD-L1 is regulated by diverse signaling in TME [155, 156]. Combinations of ICIs with chemotherapy have demonstrated efficacy in treating advanced/metastatic TNBC. They thus have been approved by the United States Food and Drug Administration (FDA) [157–160]. Progranulin (PGRN), a multifunctional growth factor expressed by breast cancer cells, can induce immune escape via upregulating PD-L1 expression on TAMs and promoting CD8^+^ T-cell exclusion [161]. The secretion of miR‐27a‐3p‐loaded exosomes from breast cancer cells, induced by endoplasmic reticulum stress, stimulates the PD‐L1 in macrophages and promotes immune evasion of breast cancer cells by activating the PTEN‐AKT/PI3K pathway [162]. The decreased expression of N-Myc downstream-regulated gene 2 (NDRG2) in breast cancer can upregulate PD-L1 expression of breast cancer cells and inhibit the proliferation of T cells [163]. Mouse UL16 binding protein-like transcript 1 (Mult1) is highly expressed in breast cancer cells carrying p53, which can bind to NK group 2 member D receptors (NKG2DRs) on NK cells, leading to NK activation and increased IFN-γ production, thus enabling the immune-eradication of cancer cells [164]. Previous studies mainly focused on the composition of immune cells in tumors. Still, more scholars believe that the spatial architecture of immune cells can affect tumor immunity and response to treatment, such as the spatial distribution of immune cells in tumors, the distance between immune cells, and other cells in the TME. [165].

### Breast cancer cells—normal epithelial cells

Breast cancer originates from epithelial progenitor cells in the breast [166]. Therefore, breast epithelial cells are inextricably associated with breast cancer cells. Normal mammary epithelial cells are closest to breast cancer cells in the early stages of carcinogenesis. The interaction between cancer cells and epithelial cells is dynamic; normal epithelial cells initially inhibit tumor cells. Coculture verified that normal epithelial cells could induce transcriptional activation p53 of breast cancer cells, resulting in apoptosis of these cells in which tyrosine phosphatases may be involved [167]. Furthermore, normal epithelial cells could induce p53-independent cell cycle arrest of breast cancer cells [167].

But the tumor-suppressive effect may not last throughout solid tumor development. Breast cancer cells can transform normal breast epithelial cells to exhibit cancerous properties, including enhanced proliferation and migration, EMT-related features, and loss of apical-basal polarity [168]. S100A8/A9 can mediate dynamic interaction between normal breast epithelial cells and adjacent breast cancer cells to induce reprogramming [169]. Coculture with breast cancer cells or induction of overexpression of S100A8/A9 revealed that normal epithelial cells exhibit features of EMT, and the capacity of cell proliferation, migration, colony formation, and three-dimensional sphere formation is increased [169]. Angiostatin, amphiregulin, CD14, ETL, insulin-like growth factor (IGFBP)-2, IGFBP-6, IGFBP-7, IL-6, latent TGF-β BP1, osteoprotegerin, PDGF-AA, secreted protein acidic and rich in cysteine (SPARC), thrombospondin (TSP), TIMP1, and VEGF are also involved in this process [168]. Exosomes from cancer cells containing miRNAs play a significant role in intercellular communication [170]. Breast cancer cell-derived exosomes, miR-21 and -10b, can silence the expression of PTEN and homeobox D10 (HOXD10) in normal epithelial cells to induce tumor formation in a Dicer-dependent manner [171].

The interaction between epithelial and breast cancer cells changes over time, transforming from inhibition to promotion of tumor cells, affecting tumor growth, proliferation, invasion, and metastasis, thus leading to heterogeneity in breast cancer.

### Breast cancer cells—breast cancer cells

Experiments have shown that the cooperation between breast cancer cells plays a vital role in the occurrence and development of breast cancer. This kind of cooperation can confer an advantage to breast cancer cell proliferation, invasion, and metastasis. Interestingly, neither luminal nor basal cells can generate cancers by themselves when transplanted into secondary recipient mice, whereas the combined transplantation of both cell types is highly carcinogenic [91]. The Wnt signaling pathway may play an essential role in this process. When this bi-clonal carcinoma is disturbed by Wnt blocking, basal subclones recruit allogeneic Wnt-producing cells to restore tumor growth [91]. EMT occurs in a subset of cells within primary breast cancers and has been shown to increase the growth of neighboring cancer cells [172]. For example, the DACH-Eyes absent (EYA)–Sineoculis homeobox homolog (SIX) network is involved in the initiation and progression of breast cancer, and their expression determines the prognosis of breast cancer patients [173–176]. When SIX1 is expressed in EMT cancer cells, VEGF-C is activated, and through neuropilin 2 (NRP2)/Fms-related tyrosine kinase 4 (FLT4), GLI signals in adjacent epithelial cancer cells are activated. The activation of GLI signals further promotes the growth, invasion, and metastasis of adjacent epithelial cancer cells, ultimately contributing to the progression of breast cancer [177]. Glutamine is a metabolite involved in the metabolic symbiosis between breast cancer cells. Glutamine-independent (luminal) breast cancer cells could synthesize glutamine to rescue glutamine-dependent (basal) BC cells in an in vitro coculture experiment, suggesting that the cell cooperation between basal and luminal cells in the mammary ducts may be achieved through glutamine symbiosis [178]. Hypoxic breast cancer cells can take up glucose and produce lactate, which can be taken up by aerobic breast cancer cells and catabolized to alanine and glutamate, which can then be exported to cells. Aerobic breast cancer cells have heterogeneity in the consumption capacity of lactate. Among them, those with high consumption of lactate express high monocarboxylate transporter (MCT)1/low MCT4 and reduce the use of glucose, which can leave more glucose to be used by other breast cancer cells [179]. This kind of cooperation can enable tumors to adapt to environmental changes and promote the generation of heterogeneity through the symbiosis of nutrients under different oxygen concentrations or nutrient states.

However, aerobic breast cancer cells with low consumption of lactate, with low MCT1/high MCT4 expression, consume less lactate and use relatively more glucose, which leads to the reduction of glucose available to hypoxic tumor cells [179]. Nutritional competition is a way in which cells compete. There are other aspects of cell competition, and many genes are thought to play essential roles in this process. MYC is one of the most commonly mutated genes in breast cancer [180], showing a strong correlation with cell competition [181]. MYC contributes to the generation of more competitive phenotypes; higher c-MYC breast cancer cells have higher numbers when cocultured with lower c-MYC breast cancer cells [182]. Higher expression of JNK, related to apoptosis, was observed in loser cells [182]. The alteration of human giant larvae homolog 1 (HUGL1) on breast cancer cell membrane can relieve the inhibition of Yes-Associated Protein (YAP), thus activating the transcription and expression of c-MYC and promoting the occurrence of Caspase-3 dependent apoptosis of adjacent cells with lower c-MYC expression, which leads to promotion of breast cancer cells with high expression of c-MYC to outcompete other cells in cell competition [183]. When breast cancer cells express high octamer-binding transcription factor 4 (Oct4), they also show inhibition to breast cancer cells with low Oct4 expression, similar to c-MYC [184]. This inhibition is even more potent when cells overexpress Oct4 and c-MYC simultaneously [184]. During this process, overexpression of Oct4 and c-MYC can induce higher expression of enolase 1 (Eno1), heat shock protein 90 α family class b member 1 (HSP90AB1), eukaryotic elongation factor 2 (Eef2), vinculin (VCL), Trail, and p53 to suppress other breast cancer cells [184]. During this process, the expression of PD-L1 and lysine demethylase 3A (Kdm3a) is downregulated and that of CD44 is upregulated in suppressed cells [184]. Breast cancer cells with higher MYC expression are more stem-like, which is regulated by MAPK/ERK. A higher proportion of MYC-positive cells in tumors significantly correlated with a short patient survival [185]. Thus, this c-MYC-mediated cellular competition helps eliminate the weaker cells to provide space and nutrition for the stronger ones, which may promote the emergence of more malignant phenotypes, heterogeneity, and progression of tumors, either naturally or during treatment. It was verified that the plasmacytoma variant translocation 1 (PVT1) promoter could inhibit MYC expression via promoter competition [186]. Furthermore, disruption of the PVT1 promoter by CRISPR can enhance breast cancer cell competition promoted by MYC [186]. The Flower is a downstream factor of MYC [96]. Breast cancer cells expressing Win human Flower isoforms (hFWE2 and hFWE4) have a competitive advantage over cells expressing Lose human isoforms (hFWE1 and hFWE3), contributing to the proliferation of winners and the cell death of losers [187]. Furthermore, breast cancer cells predominantly express the Win isoforms, whereas the stroma predominantly expresses the Lose isoforms, which may result in tumor growth and metastasis [187]. The activation of KRAS leads to the activation of Rac1 signaling, which can transmit the winner state of cells by downregulating contractile myosin, resulting in an increased mechanical deformability [188]. After an increase in mechanical deformability, cancer cells (winners) can engulf other cells (losers), and the engulfed cells experience cell death [188]. The competitive status of breast cancer cells is not invariable and will change or even reverse with the change of tumor living environment, such as pH and glucose concentration [100].

**Fig. 3:**
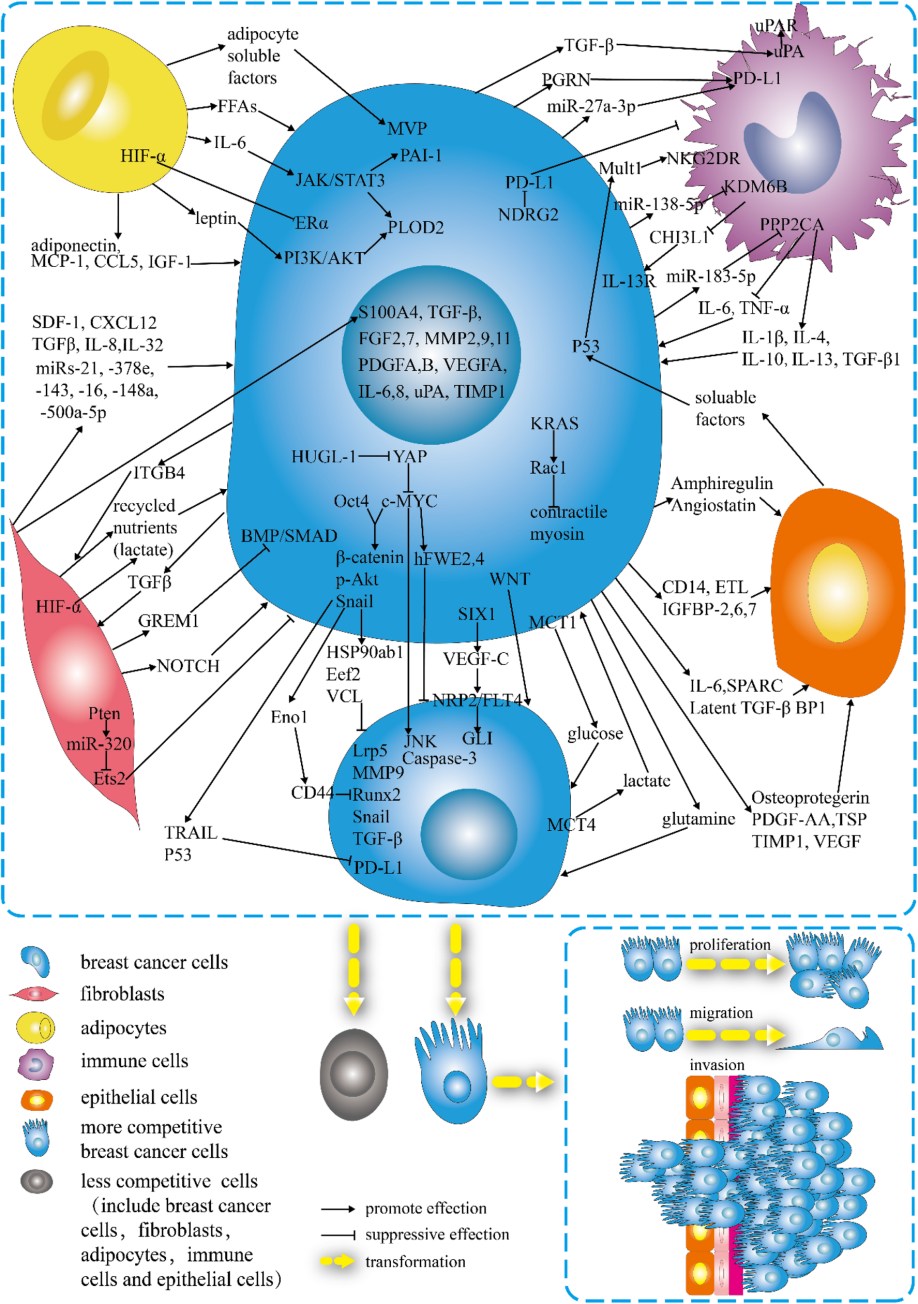
Interactions among diverse cells in the breast cancer microenvironment. The breast cancer microenvironment includes fibroblasts, adipocytes, immune cells, epithelial cells, and breast cancer cells. The interactions can be promoted (cooperative) or inhibited (competitive), leading to the generation of two cell types. The two types of cells undergo different fates, one more competitive, with more diverse and intense biological behavior, and the other gradually dying off

The interaction of breast cancer cells with TME was illustrated (Fig. 3). Indeed, many other mechanisms can explain the heterogeneity of breast cancer, such as the differentiation state of the cell of origin [98], cell plasticity, and CSC theory [189], genetic evolution of cancer, and multitask evolution [190, 191]. These mechanisms affect heterogeneity in different ways, each with advantages and shortcomings. Cell interaction provides a more comprehensive explanation of mechanisms, especially cell competition. Other mechanisms are involved in this process; for example, competition based on glycometabolism and lipid metabolism need to be explored intensively.

## Research methods to measure heterogeneity

Given that breast cancer cells exhibit heterogeneity during the evolution of mechanisms of disease progression and therapeutic evasion routes, it is critical to monitor and quantify tumor heterogeneity during diagnosis, treatment, and possibly disease resistance. Heterogeneity is present at all tumor tissue levels, so its quantification must be measured at multiple scales.

### Measuring heterogeneity in mixed cellular populations

Sequencing is one of the most used tools to measure heterogeneity [192, 193]. Since sequencing is an innovative technology in a constantly evolving field [9, 194], understanding of tumor heterogeneity is growing. Sanger sequencing is a typical example of first-generation sequencing at the genomic level. Sanger sequencing involves amplification of the DNA sequence, random termination by di-deoxy nucleotides, and fragments by their molecular weight [194]. At the transcriptomic level, RNA sequencing is often applied by the same sequencing platforms used to sequence DNA [195]. At the epigenomic level, nucleosome occupancy and methylome sequencing (NOMe–seq) based on nanopore sequencing, using an exogenous M. CviPI GpC methyltransferase, enables determination of phased patterns of native CpG methylation and chromatin accessibility [196]. As sequencing of the genome, epigenome, and transcriptome does not indicate fundamental biological processes, there was a pressing need for the sequencing of a whole protein.

At the proteomic level, in addition to traditional techniques, such as chromatography-based techniques, ELISA, and western blotting, many advanced methods have appeared, such as protein microarray, gel-based approaches, mass spectrometry (MS), Edman sequencing, and other high-throughput techniques [197, 198]. In the current development trend, the method of multi-omics combination will be a hot spot. The combination of third-generation DNA sequencing and proteomics is a prime example; the approximate shape, volume, charge, rotational diffusion coefficient, and dipole moment of individual proteins can be determined by zeptolitre sensing volume of bilayer-coated solid-state nanopores [199].

### Measuring heterogeneity in single cells

Though it is insufficient, most of the above analyses are based on the analysis of tissue lysates, which hinders the cellular origin of the analyzed molecules, and some of the information of the smaller cell subsets is lost, affecting the diagnosis, treatment, and prognosis of tumors. A better understanding of genetic differences within cells is urgent and will lead to a better understanding of the heterogeneity of breast cancer. Based on second-generation sequencing, single-cell sequencing has progressed rapidly in recent years, revolutionizing our ability to drill down into thousands of individual cells' genomic, transcriptomic, epigenomic, and metabolic properties to enable an unbiased analysis of cells in tumoral lesions [200]. At the genomic level, the highly multiplexed single nucleus sequencing (HM-SNS) has been demonstrated as a good tool to explore the heterogeneity of copy number profiling in the breast cancer [201], which has been applied to explain the chemoresistance revolution in TNBC [16]. Deger et al. reported a modular pipeline constructed of five individually adjustable steps, including enrichment, isolation, whole-genome amplification, low pass sequencing, and bioinformatical analysis, to perform single CTCs genomics at a high resolution by copy number profiling [60]. Each step can be adjusted to use different methods according to specific research needs. Furthermore, it is a minimally invasive method to monitor molecular changes occurring during disease progression. At the epigenomic level, detecting multiallelic copy number events and copy-neutral loss-of-heterozygosity by single-cell transposase-accessible chromatin sequencing (scATAC-seq) enables the analysis of the contribution of chromosome instability and chromatin remodeling to tumor evolution [10]. Single-cell deoxyribonuclease I digestion sequencing (scDNase-seq) [11] and single-cell NOMe–seq (scNOMe-seq) [12] have emerged but are still rarely used at present. Due to its advantages in the field of epigenetics, it is believed to bring great convenience and breakthrough in the study of the heterogeneity of breast cancer shortly. Single-cell chromatin immunoprecipitation followed by sequencing approach (scChIP-seq), a high-throughput droplet microfluidics platform, paves the way to study the role of chromatin heterogeneity [202]. At the transcriptomic level, single-cell RNA sequencing (scRNA-seq) involves the capture of mRNA from compartmentalized cells, reverse transcription into cDNA, amplification, and identification by oligomeric indexes and molecular markers enabling high-throughput analysis of transcriptome [203]; scRNA-seq has been used widely in exploring heterogeneity of breast cancer, including metabolism [204], characterizing heterogeneous subgroups of different subtypes of breast cancer [205, 206], cell cycle [207], EMT [208], CTCs [209], and cell interaction [209]. At the proteomic level, the development of microfluidic imaging flow cytometry, which utilizes high-speed imaging technology, has facilitated the high spatial and temporal resolution of protein expression in individual cells. For example, an optofluidic flow cytometer (OFCM) combines a multistage microfluidic chip and a four-color fluorescence detection system. Single-cell phenotypic analysis can be done on 1.2 mL of whole blood per hour with OFCM and CTC separation, 3D focusing in the microchannel, and counting in real time [210]. Liquid chromatography - quadrupole time-of-flight tandem mass spectrometer (LC-Q-TOF-MS/MS) can achieve an extremely high degree of separation by LC; in addition, it provides information on the structure of flavonoids by Q-TOF-MS/MS [211], which has been applied to reveal the mechanism of treatment of TNBC [212].

### Measuring heterogeneity in tissue slices preserving spatial information

Single-cell analysis can result in a loss of spatial information. To overcome this obstacle, the appearance of spatial omics has improved the imaging capabilities to produce subcellular spatial visualization and quantitative analysis of breast cancer tissue. At the genomic level, by combining laser-capture micro-dissection, laser catapulting, whole-genome amplification, and single-cell DNA sequencing, topographic single-cell sequencing (TSCS) can be used to measure genomic copy number profiles while preserving spatial information in the tissue slices. TSCS has revealed that one or more clones escape the ducts and migrate into the adjacent tissues to establish invasive carcinomas [213]. At the epigenomic level, the transposome-assisted single nucleus barcoding approach for ATAC-seq (SNuBar-ATAC) can easily label and multiplex a large number of samples together for parallel sequencing in a single microdroplet experiment with spatial information in breast cancer [214]. It has also been used in studying chromatin accessibility changes induced by drug treatment combinations. At the transcriptomic level, spatial transcriptomics provides quantitative gene expression data and visualization of mRNA distribution in tissue slices by locating tissue slices on aligned reverse transcription primers using unique location barcodes [215]. For miRNA analysis, fluorophore-encoded error-corrected labels (FluoELs) have low cytotoxicity and are capable of quantifying and spatially resolving breast cancer-related miRNAs and evaluating their coordination. For error-corrected quantification, FluoELs combine proportional dual fluorophores with a constant quantitative single fluorophore [216]. Cellular indexing of transcriptomes and epitopes by sequencing (CITE-seq) can spatially characterize high-resolution immunophenotypes, including new PD-L1/PD-L2+ macrophage populations associated with clinical outcomes [19]. At the proteomic level, multiplexed ion beam imaging by the time-of-flight (MIBI-TOF) couples bright ion sources and orthogonal TOF-MS to image metal-tagged antibodies at the subcellular level [217] and has been used to explore the TME structure. MIBI-TOF verified that myoepithelial disruption could be a protective factor against recurrence [218]. Imaging mass cytometry, which couples immunohistochemical and immunocytochemical methods with high-resolution laser ablation to single-cell cytometry by TOF, reserving spatial information and delineating cell subpopulations and cell interactions in breast cancer [219]. High-resolution atlas of breast cancer has been well established, such as the atlas of CAFs [220], immune ecosystem [221, 222], and the interaction of these components with spatial information [223].

### Measuring heterogeneity in patients through medical imaging

In addition to the level of tumor cells or tissues, the study of tumor heterogeneity on the patient level is important. Imaging techniques, including mammography, ultrasound (US), and magnetic resonance imaging (MRI) are conventional and effective tools for measuring the heterogeneity of breast cancer patients. Nevertheless, several limitations are associated with these techniques, such as high cost, harmful radiation, lack of sensibility, and inconvenience to the patients [224]. Positron-emission tomography (PET) and single-photon emission computed tomography (SPECT) are useful imaging techniques for the detection of metabolism and metastasis in breast cancer patients, but there is a lack of anatomical details in the imaging [225]. Thus, the combination of several techniques is recommended [226], such as PET/MRI and SPECT/MRI. However, these classical imaging methods, including mammography, US, MRI, PET, and SPECT, are primarily qualitative and subjective in tumor evaluation. Analyses based on tumor biopsies provide limited data on tumor characteristics because the sample extracted may not always represent the heterogeneity of the entire patient's tumor [227]. Due to the need for a quantitative and whole heterogeneity evaluation of breast cancer, radiomics has been gradually used in this field. Radiomics begins with obtaining high-quality images from mammography, US, MRI, PET, etc., and from these images, identifies an area of interest. They are segmented with operator edits and eventually rendered in three dimensions. In addition to clinical and genomic data, the quantitative features are extracted to generate reports for storage in the database. Then, these data are mined, which can be done using artificial intelligence, machine learning, or statistical approaches. Then, diagnostic, prediction, or prognostic models are established to obtain results [228]. The application of radiomics in breast cancer has been well reviewed [229, 230], and it will be possible to improve the diagnostic accuracy of breast imaging and increase the accuracy of heterogeneous studies by adding radiomics to the standard radiology workflow.

In conclusion, the quantification of heterogeneity of breast cancer needs to be measured at multiple scales. Each method has its advantages and disadvantages (Table 1). Depending on the actual situation, choosing the appropriate single or combined approach is essential.

**Table 1:** Advantages and disadvantages of various research methods to measure heterogeneity

State of sample	Application	Example	Advantages	Disadvantages	Refs.
Mixed cellular populations	At genomic and transcriptomic levels	Sanger sequencing	High accuracy	Laborious, expensive, lack of single-cell information and spatial information	[195]
	At the epigenomic level	NOMe–seq	Single-molecule real-time sequencing	Limited by the incompleteness of the human genome reference, with large gaps persisting in highly repetitive areas	[196]
	At the proteomic level	Chromatography-based techniques, ELISA, western blotting, protein microarray, gel-based approaches	Identification of biomolecules using inherent properties, sequence of molecules, electrical charge	High effects of low quality and/or quantity of biomolecules. Limited in identification of rare peptides	[9, 197]
		MS	Label-free. High throughput. Identification of posttranslational modifications	High complexity of analysis. Limited in identification of rare peptides	[9, 197]
		Edman sequencing	Useful for the elucidation of residue deletions, the presence of common stable derivatives, and for following the progress of the synthesis itself	Limited in quantitation and the type and degree of adduct formation	[197, 231]
Single cells	At the genomic level	HM-SNS	Preserving single-cell information. High throughput	Loss of spatial information	[16, 201]
		Modular single CTC analysis pipeline	Each step is adjustable. Preserving single-cell information	Loss of spatial information	[60]
	At the epigenomic level	scATAC-seq	Preserving single-cell information	Loss of spatial information	[10]
		scDNase-seq	Preserving single-cell information	Loss of spatial information	[11]
		scNOMe–seq	Preserving single-cell information	Loss of spatial information	[12]
		scChIP-seq	Preserving single-cell information. High throughput	Chip design is highly complex. Loss of spatial information	[202]
	At the transcriptomic level	scRNA-seq	Preserving single-cell information	Loss of spatial information	[203–209]
	At the proteomic level	OFCM	Preserving single-cell information. Counting in real time	Loss of spatial information. Chip design is highly complex	[210]
		LC-Q-TOF-MS/MS	Preserving single-cell information	Loss of spatial information	[211, 212]
Tissue slices	At the genomic level	TSCS	Preserving single-cell information. Preserving spatial information	High complexity in use	[213]
	At the epigenomic level	SNuBar-ATAC	Preserving single-cell information. Preserving spatial information. Highly accurate. Easy to use. High throughput	Loss of partial sequencing reads. Mapping rate is not very high	[214]
	At the transcriptomic level	FluoELs	Preserving single-cell information. Preserving spatial information. Error-correcting capability. Low cytotoxicity	Low throughput	[216]
		CITE-seq	Preserving single-cell information. Preserving spatial information	Loss of information of some cell types	[19]
	At the proteomic level	MIBI-TOF	Preserving single-cell information. Preserving spatial information. Allow to revisit a sample after prolonged periods of time		[217]
		IMC	Preserving single-cell information. Preserving spatial information	Antibodies are not commercially available	[9, 219]
Organs/patients		Mammography, US, and MRI	Relatively effective and widely adapted in hospitals	Excessive cost. Harmful radiation. Lacks sensibility. Inconvenience to the patients	[224]
		PET, SPECT	Effective in detection of metabolism and metastasis	Anatomical details are lacking	[225]
		Radiomics	Contain first-, second-, and higher-order statistics. Possibility of data sharing. Ability of quantitation	Difficulty in reproducibility	[228]

CITE-seq, cellular indexing of transcriptomes and epitopes by sequencing; CTCs, Circulating tumor cells; HM-SNS, highly multiplexed single nucleus sequencing; FluoELs, fluorophores encoded error-corrected labels; IMC, imaging mass cytometry; LC-Q-TOF-MS/MS, liquid chromatography-quadrupole time-of-flight tandem mass spectrometer; MIBI, multiplexed ion beam imaging; MRI, magnetic resonance imaging; NOMe–seq, nucleosome occupancy and methylome sequencing; MS, mass spectrometry; OFCM, optofluidic flow cytometer; PET, Positron-emission tomography; TSCS, topographic single-cell sequencing; scATAC-seq, single-cell transposase-accessible chromatin sequencing; scDNase-seq, single-cell DNase sequencing; scNOMe–seq, single-cell  NOME-seq; scRNA-seq, single-cell RNA sequencing; SNuBar-ATAC, single nucleus barcoding approach for transposase-accessible chromatin sequencing; SPECT, single-photon emission computed tomography; US, ultrasound

## Breast cancer heterogeneity and precise treatment

As mentioned above, considering heterogeneity, precise breast cancer treatments should be based on genetic, transcriptomic, epigenetic, proteomic, biomarkers, metabolism, cell cycle, TME, EMT, CTCs, and clinical and histopathological aspects. There are many standard treatment schemes that have been widely adopted in clinical practice based on breast cancer heterogeneity. Surgery, radiotherapy, and chemotherapy are standard therapies for all subtypes of breast cancer [232]. Standard breast surgery is either mastectomy, which means a complete excision of the breast, usually followed by reconstruction, or lumpectomy, which preserves the breast [233]. Axillary is the removal of the axillary lymph nodes to help determine the spread of cancer cells and remove the cancer cells [233]. Radiotherapy refers to high-energy radiation applied to the whole or part of the breast (after lumpectomy), the chest wall (after mastectomy), and regional lymph nodes [234]. A short course of radiotherapy of 3–4 weeks is usually as effective as a long course. Chemotherapy generally includes anthracyclines, taxanes, antimetabolites, alkylating agents, platinum drugs, and vinca alkaloids [235]. Chemotherapy can be further defined into NAC (before surgery) and adjuvant chemotherapy (after surgery). NAC, which often includes targeted agents, is a standard of care for non-metastatic but inoperable breast cancer and can help to reduce and adjust risk before or after breast and axillary surgery [232]. Different subtypes of tumors have varied sensitivities to neoadjuvant chemotherapy, possibly due to different immunological infiltrate biology. Increased TIL concentrations are positively associated with better response to neoadjuvant chemotherapy in HER2^+^ breast cancer and TNBC. However, increased TILs is negatively associated with better response to NAC in luminal-HER2^–^ breast cancer [236]. Adjuvant chemotherapy is used in breast cancer patients with lymph node metastases or at high risk for recurrence [237]. As with neoadjuvant chemotherapy, ER^–^ breast cancer patients have lower recurrence and mortality rates than ER^+^ breast cancer patients after adjuvant chemotherapy [238]. Surgery, radiotherapy, and chemotherapy are common strategies for treating patients with BC. However, because patients respond differently to radiotherapy or chemotherapy, they are not effective enough to treat all BC molecular subtypes, so personalized treatment is essential.

Cyclin-dependent kinase (CDK) 4 and CDK6 inhibitors, PI3K inhibitors, polyadenosine-diphosphate-ribose polymerase (PARP) inhibitors, and anti-PD-L1 immunotherapy are examples of standard treatment options for metastatic breast cancer [232].

### Personalized precision therapy for HER2^+^ breast cancer

As mentioned above, patients are usually managed based on the ER/PR/HER2 status of the cancer [233, 239]. As for early HER2^+^ breast cancer treatment, NAC includes trastuzumab plus pertuzumab plus chemotherapy (including taxanes, with or without anthracyclines or platinum drugs such as carboplatin) and is recommended before surgery [232]. Trastuzumab (Herceptin), a humanized monoclonal antibody targeting HER2, was the first biological drug-approved therapy and gold standard for HER2^+^ breast cancer [240]. Trastuzumab binds to subdomain IV of the HER2 extracellular domain [241]. Pertuzumab (Perjeta) binds to subdomain II of the HER2 extracellular domain to function [242]. After surgery, if patients have achieved pathological complete response (pCR), trastuzumab with or without pertuzumab is recommended for 12 months. If not, trastuzumab emtansine (T-DM1) is recommended for 14 cycles [232]. T-DM1 is administered because HER2 heterogeneity makes anti-HER2 therapy alone insufficient to eradicate HER2^+^ cancer completely. It is an antibody–drug conjugate composed of an anti-HER2 antibody and a microtubule inhibitor [243], showing high targeting ability and an excellent therapeutic effect on metastasis in several clinical trials [244, 245].

For metastatic/advanced HER2^+^ breast cancer, trastuzumab plus pertuzumab plus docetaxel or paclitaxel are recommended as the first-line therapy [232]. Second-line therapy consists of T-DMI. However, its role in eliminating HER2 heterogeneity is limited, with no pCR observed among patients with HER2 heterogeneity [246]. Trastuzumab deruxtecan (DS-8201) is another antibody–drug conjugate composed of an anti-HER2 antibody and a potent topoisomerase I inhibitor [247]. Its drug-antibody ratio is higher than T-DM1 while maintaining good pharmacokinetic characteristics [247]. DS-8201 has also been shown to play a therapeutic role in HER2^+^ metastatic breast cancer in several clinical trials [248, 249]. Furthermore, DS-8201 has been demonstrated to have a bystander-killing effect, which means deruxtecan is released into the spaces between cells and eliminates HER2^–^ tumor cells after DS-8201 kills HER2-positive cancer cells to overcome intratumor heterogeneity, both in vitro and in vivo [250]. Trastuzumab duocarmazine (SYD985), which contains an anti-HER2 antibody, and duocarmycin [251], which can reduce the number of cancer cells through DNA damage and bystander-killing effects [251, 252], can be used to overcome intratumor heterogeneity. Its efficacy and safety have been demonstrated in a phase I study [253].

### Personalized precision therapy for HR^+^ breast cancer

For the treatment of HR^+^/HER2^–^ breast cancer, endocrine therapy, such as selective ER modulators (SERMs), selective ER deregulators (SERDs), and aromatase inhibitors (AIs), are mainstream. SERMs, such as tamoxifen, toremifene, bazedoxifene, and raloxifene, are antiestrogens that can competitively bind to the ER and render estrogen inactive [233]. SERDs, such as fulvestrant, once bound to the ER, inhibit receptor dimerization and prevent the ER's translocation to the nucleus, leading to its degradation and, in contrast to SERMs, completely block the ER signaling pathway [254]. AIs can be divided into two categories: steroidal AIs and non-steroidal AIs, which can block aromatase activity, inhibiting estrogen synthesis [255]. After surgery or radiotherapy, endocrine therapy is recommended for early-stage HR^+^ breast cancer. For postmenopausal women, tamoxifen is recommended, or AIs if contraindicated. For premenopausal women, ovarian function inhibitors are added [232, 233].

For metastatic/advanced HR^+^ breast cancer, a CDK4/6 inhibitor combined with endocrine therapy should be considered a standard of care [256]. For example, the combination of a CDK4/6 inhibitor named ribociclib and AIs, or tamoxifen, has better efficacy than endocrine therapy alone [257]. Another first-line treatment for advanced ER^+^ breast cancer is fulvestrant monotherapy [258]. Fulvestrant alone has no significant disadvantage compared with the combination of other endocrine therapies [233]. When treating patients with PIK3CA-mutant advanced HR^+^ tumors, a PIK3 inhibitor named alpelisib combined with fulvestrant is recommended, which can improve median overall survival by about eight months than fulvestrant alone [259]. PARP is a damage recognition repair protein of single-strand break, which plays a vital role in initiating the repair of the single-strand break. Inhibition of PARP leads to the accumulation of single-strand breaks and the formation of double-strand breaks.

BRCA-mutated cells cannot repair double-strand breaks, eventually leading to cell death [260]. When treating patients with BRCA1 or BRCA2-mutant advanced HR^+^ tumors, PARP inhibitors, such as olaparib or talazoparib, are recommended [261, 262]. Antibody-drug conjugates targeting the ER receptor are less well studied, among which Proteolysis-Targeting Chimeras (PROTACs) targeting ER are currently attracting attention in medicinal chemistry [263]. PROTACs are degrader-antibody conjugates that bind both an E3 ligase and a protein of interest and work by forming a ternary complex that initiates protein of interest ubiquitination and degradation by hijacking the ubiquitin–proteasome system [264]. ARV-471, a PROTAC targeting ERα, has entered the clinical phase II trial [265], has prominent antitumor activity, and reduces ER protein expression by more than 90% in cell lines and 62% in tumor tissues. Furthermore, several new PROTACs are undergoing experimental assessment [263]. The studies of PROTACs will make a breakthrough contribution to overcoming drug resistance in ER^+^ breast cancer in the future. As for HR^+^/HER2^+^ patients, a combination of hormone therapy and targeted anti-HER2 approaches, which have been shown to control the disease over the long term, is preferable [32].

### Personalized precision therapy for triple-negative breast cancer

The conventional treatment mode of TNBC, known for its challenges in therapy with intratumor heterogeneity, involves a combination of surgery, radiotherapy, and chemotherapy. Despite the emergence of new biological and targeted agents, the primary treatment for TNBC remains cytotoxic chemotherapy. As for the treatment of early TNBC breast cancer, chemotherapy (including adriamycin, cyclophosphamide, and paclitaxel, with or without carbo platinum) is recommended before surgery [232, 266]. After surgery, if patients have achieved pCR, they need only be followed up regularly. If not, these patients should consider capecitabine, an oral prodrug of fluorouracil, for 6–8 courses [232]. There are clinical trials that show that adjuvant chemotherapy with capecitabine after standard neoadjuvant chemotherapy containing anthracyclines, taxanes, or both is safe and effective in prolonging disease-free survival and overall survival in patients with TNBC who have residual invasive disease on pathological testing [267].

For metastatic/advanced TNBC breast cancer, patients should complete testing for a germline BRCA mutation and PD-L1 before treatment [232]. Because of remarkable genomic instability and increased immune infiltration, certain TNBCs display high PD-L1 expression compared with other subtypes [268]. These properties make patients with TNBC good candidates for ICIs such as atezolizumab and pembrolizumab. Therapeutic blockade of PD-L1 with atezolizumab [269] or therapeutic blockade of PD-1 with pembrolizumab [270] can activate and enhance tumor-specific T-cell responses, resulting in increased antitumor activity. In a phase I study of 111 patients with TNBC, the PD-1 inhibitor pembrolizumab [157] demonstrated therapeutic effects and acceptable safety. If patients are PD-L1^+^, nab-paclitaxel plus atezolizumab or paclitaxel plus pembrolizumab are recommended [232]. If patients are BRCA mutation-positive, PARPi are recommended [232] as examples of PARPi, olaparib and veliparib are being evaluated in clinical experiments [271, 272]. If patients are PD-L1^–^ or BRCA wild-type, combination chemotherapy or sequential single-agent chemotherapy should be considered, including anthracycline or taxanes. If these drugs are not available, carboplatin, eribulin, vinorelbine, capecitabine, and sacituzumab govitecan are recommended [232]. Sacituzumab govitecan is a type of antibody-drug conjugate that combines a topoisomerase I inhibitor SN-38 and an antibody targeting trophoblast antigen 2 (Trop-2) [273]. In a randomized, phase III trial, among patients with metastatic TNBC, progression-free survival and overall survival were about twice as long in those treated with sacituzumab govitecan as in those treated with single-agent chemotherapy [274]. Vaccination is an emerging approach to stopping the recurrence of TNBC in high-risk patients. The individual selection of vaccine antigens from a set of different candidate peptides based on the pre-existing host immunity is known as personalized peptide vaccination [275]. In a phase II study of personalized peptide vaccination for metastatic recurrent breast cancer patients, personalized peptide vaccination was reported to have limited adverse events and boost cytotoxic T lymphocyte or IgG response in patients, especially TNBC patients [275].

The precise treatment of each subtype of TNBC is still in its infancy. LAR is characterized by high expression of androgen receptors, which can be a target for treatment. Bicalutamide, an androgen receptor (AR) agonist, was evaluated in a multicenter phase II trial. The results showed that the 6-month clinical benefit rate was 19% for bicalutamide, and the median progression-free survival was 12 weeks with no grade 4/5 treatment-related adverse events observed [276]. Enzalutamide and abiraterone are androgen receptor inhibitors, showing significant clinical activity and tolerability in patients with locally advanced or metastatic AR^+^ TNBC [277, 278]. Furthermore, 70% of LAR tumors exhibit somatic mutations in the PI3K signaling pathway; thus, PI3K and AKT inhibitors may be beneficial [279]. LAR retained retinoblastoma tumor suppressor and showed frequent CDK inhibitor 2A gene alterations, both of which are associated with CDK4/6 inhibitors. Therefore, patients with LAR tumors may benefit from CDK4/6 inhibitors [279]. IM subtype is characterized by elevated immune cell signaling and TILs. Additionally, ICI genes such as PD1 and PD-L1 showed high expression in IM [279]. Thus, ICIs may be a potential strategy for IM. BLIS subtype is characterized by high genomic instability, suggesting that PARP inhibitors and other DNA-damaging chemotherapy can be a potential strategy for BLIS [279]. The MES subtype is characterized by overexpression of stem cell-related genes such as the STAT3 signaling pathway, suggesting that targeting the STAT3 pathway can be a potential strategy for MES [279].

### Personalized precision therapy for claudin-low breast cancer

Research on claudin-low breast cancer has gradually increased in popularity recently. The high rate of metastasis and mortality in patients with claudin-low tumors remains troubling [280] because of its aggression, chemoresistance, and lack of targeted therapies. Claudin-low tumors are characterized by high enrichment for EMT markers, immune response genes, and cancer stem cell-like features; these are potential therapeutic targets. Topsentinol L trisulfate can inhibit the activation of AMP-activated protein kinase and checkpoint kinase 1 and promote activation of p38 in claudin-low breast cancer. Topsentinol L trisulfate has higher efficacy against claudin-low breast cancer than other types [281]. Most claudin-low breast tumors are reported to be ER^–^, PR^–^, and HER2^–^; therefore, the treatment of TNBC can play a guiding role in the treatment of claudin-low breast cancer to a certain extent [30, 282].

### Personalized precision therapy targeting cell interactions

Targeting cell interactions as a therapeutic breakthrough is a promising approach. In theory, all the genes or proteins mentioned above that interact with cells have potential therapeutic implications. Currently, related drugs are gradually being used in clinical practice. AVI-4126 is a phosphorodiamidate morpholino oligomer that can inhibit MYC expression by preventing ribosomal assembly [283]. In a phase I study, the findings suggest that AVI-4126 is concentrated in breast cancer as expected and can inhibit the target [283]. TAS-119 has the potential to target the MYC and Wnt/β-catenin pathways [284], with no complete response or partial response after the use of TAS-119 in patients with MYC-amplified/β-catenin mutations [285]. Furthermore, short-term doxorubicin and cisplatin administration may induce a more favorable TME and increase the likelihood of response to PD-1 blockade in the TNBC [158]. Bevacizumab, a type of VEGF inhibitor [286, 287], and cediranib, a VEGFR1-3 inhibitor [288], have also achieved good results in breast cancer treatment. Bispecific antibodies (BsAbs) are another candidate for the effective treatment of cancer and are currently in various stages of clinical trials [289, 290]. A BsAb named M802 consists of a monovalent unit against HER2 and a single chain unit against CD3. M802 could recruit CD3^+^ immune cells to eliminate breast cancer cells. It was more cytotoxic than trastuzumab in cells with high expression of HER2, low expression of HER2, and trastuzumab resistance [291]. Ertumaxomab is a bispecific, trifunctional antibody that binds to tumor cells expressing HER2/neu, T cells expressing CD3, and accessory cells expressing Fcγ, forming a tri-cell complex [292]. In a phase I trial, Ertumaxomab showed promising efficacy and transient and reversible side effects of HER2 overexpressing cancers, including breast cancer [292]. Anti-CD3 × anti-HER2 bispecific antibody-armed activated T cells (HER2 BATs) showed promising efficacy in killing breast cancer cells expressing high levels of HER2 and low HER2 receptor expressing breast cancer cells in a non-MHC restricted manner by perforin and granzyme B [293, 294]. A phase I trial suggested that HER2 BATs can target HER2^+^ and HER2^–^ tumors and increase Th1 cytokines and IL12 against breast cancer [295]. In a phase II trial, immune consolidation (IL-2 and granulocyte–macrophage-colony stimulating factor) with HER2 BATs after chemotherapy was demonstrated to increase the proportion of patients who were stable at four months and the median overall survival in HER2^–^ metastatic breast cancer patients [293]. Unfortunately, immunotherapy for ER^+^ breast cancer has historically fared poorly, thus making ER^+^ breast cancer the most difficult subtype for BsAbs to target [296]. This will be one of the development directions of BsAbs in the future.

### Personalized precision therapy guided by single-cell sequencing

Despite significant advances in heterogeneous individualized therapy for breast cancer, with a growing list of targeted drugs in adjuvant, neoadjuvant, and metastatic therapies, the need to finetune precision drug therapy to improve survival and eliminate late relapse remains unmet. Single-cell sequencing is a powerful tool for exploring tumor heterogeneity and guiding the improvement of traditional therapies. As mentioned above, single-cell sequencing can detect cell resistance to conventional therapies to apply further novel drugs targeting these cells [73]. Single-cell RNA sequencing can be a powerful tool for discovering novel therapies associated with the immune checkpoint crosstalk [297]. Radioresistant tumor cells have a higher rate of PD-L1 positivity and tumor mutation burden, as indicated by the single-cell RNA sequencing [297]. Thus, combining immune checkpoint therapy and radiation therapy guided by single-cell RNA sequencing may be beneficial. Single-cell sequencing can also monitor changes in the genome, epigenome, transcriptome, and proteome after treatment and adjust the treatment regimen. Single-cell RNA sequencing revealed that intratumoral plasmid IL12 can expand CD8^+^ T cells, induce a *CXCR3* gene signature, and sensitize patients to anti-PD-L1 therapy [298]. A patient who had not previously responded to anti-PD-L1 therapy had increased CXCR3 after intratumoral injection of plasmid IL12. Immediately, she received additional anti-PD-1 therapy and showed a significant clinical response. Minimally invasive thermal therapy has been attempted for breast cancer and can induce an immune response. By performing single-cell RNA sequencing on peripheral blood mononuclear cells from patients before and after microwave ablation, researchers found that B cells are critical antigen-presenting cells that initiate CD4^+^ T cells in the microwave ablation-induced immune response [299]. This study provides a comprehensive picture of microwave ablation-induced systemic immune responses and opens a new way to identify and improve potential targets of immune responses. Combining single-cell sequencing with other technologies further promotes the development of personalized therapies, such as hydrosequencing, which can analyze ER, PR, HER2 expression, and other clinical markers of CTCs from 10 mL of blood [300].

## Conclusion

We summarized the current state of knowledge on the origins of breast cancer heterogeneity and highlighted cell interactions in this process. We have reviewed different levels of breast cancer heterogeneity and described its enormous complexity. In particular, we have defined new research methods, a possible mechanism of cell interaction, and clinical treatment progress. Cell interactions have a unique advantage in explaining the genesis of heterogeneity and further treatment of breast cancer. This theory treats cells as individuals and endows them with characteristics and behaviors such as cooperation and competition between cells, just like individual organisms in nature, which is appropriate and reasonable. Targeting the heterogeneity of breast cancer at different levels, intercellular interactions, supported by single-cell sequencing and spatial transcriptome, will be the focus of future research and treatment.

## Data Availability

Not applicable.

## Abbreviations

TME: Tumor microenvironment
EMT: Epithelial–mesenchymal transition
CTCs: Circulating tumor cells
ER: Estrogen receptor
PR: Progesterone receptor
HRs: Hormone receptors
HER2: Human epidermal growth factor receptor 2
TNBC: Triple-negative breast cancer
LAR: Luminal androgen receptor
MES: Mesenchymal-like
BLIS: Basal-like and immune suppressed
CSCs: Cancer stem cells
BCSCs: Breast cancer stem cells
TNM: Tumor, lymph node, metastasis
VM: Vasogenic mimicry
NAC: Neoadjuvant chemotherapy
SERMs: Selective ER modulators
SERDs: Selective ER deregulators
AIs: Aromatase inhibitors
TILs: Tumor-infiltrating lymphocytes
NES: Neutrophil-enriched subtype
MES: Macrophage-enriched subtype
CCR2: C–C chemokine receptor type 2
ICB: Checkpoint blockade
CAFs: Cancer-associated fibroblasts
SDF-1: Stromal-derived factors
CXCL12: C-X-C motif ligand 12
TGFβ: Transforming growth factor-beta
IL-6: Interleukin-6
IL-8: Interleukin-8
HIF-α: Hypoxia-inducible factor-α
S100A4: S100 calcium-binding protein A4
FGF2: Fibroblast growth factor 2
FGF7: Fibroblast growth factor 7
MMP2: Matrix metalloproteinase 2
MMP9: Matrix metalloproteinase 9
MMP11: Matrix metalloproteinase 11
PDGFA: Platelet-derived growth factor A
PDGFB: Platelet-derived growth factor B
VEGFA: Vascular endothelial growth factor A
uPA: Urokinase-type plasminogen activator
TIMP1: Tissue inhibitor matrix metalloproteinase 1
BMP: Bone morphogenetic protein
GREM1: GREMLIN1
miR: microRNA
PTEN: Phosphatase and tensin homolog
ITGB4: Integrin β 4
CAAs: Cancer-associated adipocytes
JAK: Janus kinase
STAT3: Signal transducer and activator of transcription 3
PI3K: Phosphoinositide 3-kinases
AKT: Protein kinase B
PLOD2: Lysyl hydroxylase
IL-1β: Interleukin-1 beta
TNF-α: Tumor necrosis factor α
PAI-1: Plasminogen activator inhibitor-1
FFAs: Free fatty acids
MCP-1: Monocyte chemoattractant protein-1
CCL5: CC-chemokine ligand 5
IGF-1: Insulin-like growth factor 1
MVP: Major vault protein
TAMs: Tumor-associated macrophages
IL-4: Interleukin-4
IL-10: Interleukin-10
IL-13: Interleukin-13
uPAR: Urokinase receptor
KDM6B: Lysine demethylase 6B
CHI3L1: Chitinase 3-like protein 1
IL-13Rα2: Interleukin-13 receptor α2 chain
MAPK: Mitogen-activated protein kinase
PPP2CA: Protein phosphatase 2 catalytic subunit alpha
NK: Natural killer
PD-1: Programmed death protein 1
ICIs: Immune checkpoint inhibitors
FDA: Food and Drug Administration
PGRN: Progranulin
NDRG2: N-myc downstream-regulated gene 2
Mult1: Mouse UL16 binding protein-like transcript 1
NKG2DRs: Natural killer group 2 member D receptors
S100A8: S100 calcium-binding protein A8
S100A9: S100 calcium-binding protein A9
IGFBP: Insulin-like growth factor
SPARC: Secreted protein acidic and rich in cysteine
TSP: Thrombospondin
HOXD10: Homeobox D10
DACH: Diaminocyclohexane
EYA: Eyes absent
SIX1: Sineoculis homeobox homolog 1
NRP2: Neuropilin 2
FLT4: Fms-related tyrosine kinase 4
MCT: Monocarboxylate transporter
HUGL1: Human giant larvae homolog 1
YAP: Yes-associated protein 1
Oct-4: Octamer-binding transcription factor 4
Eno1: Enolase 1
HSP90AB1: Heat Shock Protein 90 Alpha Family Class B Member 1
Eef2: Eukaryotic elongation factor 2
VCL: Vinculin
Kdm3a: Lysine demethylase 3A
PVT1: Plasmacytoma variant translocation 1
hFWE: Human Flower isoform
NOMe–seq: Nucleosome occupancy and methylome sequencing
MS: Mass spectrometry
HM-SNS: highly multiplexed single nucleus sequencing
scChIP-seq: Single-cell chromatin immunoprecipitation followed by sequencing
scATAC-seq: Single-cell transposase-accessible chromatin-sequencing
scDNase-seq: Single-cell DNase sequencing
scNOMe–seq: Single-cell NOMe–seq
scRNA-seq: Single-cell RNA sequencing
OFCM: Optofluidic flow cytometer
LC-Q-TOF-MS/MS: Liquid chromatography - quadrupole time-of-flight tandem mass spectrometer
TSCS: Topographic single cell sequencing
SNuBar-ATAC: Single nucleus barcoding approach for transposase-accessible chromatin-sequencing
FluoELs: Fluorophores encoded error-corrected labels
CITE-seq: Cellular indexing of transcriptomes and epitopes by sequencing
MIBI-TOF: Multiplexed ion beam imaging by time of flight
IMC: Imaging mass cytometry
US: Ultrasound
MRI: Magnetic resonance imaging
PET: Positron-emission tomography
SPECT: Single-photon emission computed tomography
T-DM1: Trastuzumab emtansine
pCR: Pathologic complete response
CDK: Cyclin-dependent kinase
PARP: Polyadenosine-diphosphate-ribose polymerase
DS-8201: Trastuzumab deruxtecan
PROTACs: Proteolysis-targeting chimera
SYD985: Trastuzumab duocarmazine
Trop-2: Trophoblast antigen 2
AR: androgen receptor
BsAb: Bispecific antibody
