# Correction: Breast cancer heterogeneity and its implication in personalized precision therapy

**DOI:** 10.1186/s40164-024-00472-z

**Published:** 2024-01-23

**Authors:** Liantao Guo, Deguang Kong, Jianhua Liu, Ling Zhan, Lan Luo, Weijie Zheng, Qingyuan Zheng, Chuang Chen, Shengrong Sun

**Affiliations:** 1 Department of Breast and Thyroid Surgery, Renmin Hospital of Wuhan University, No. 238 Jiefang Road, Wuchang District, Wuhan, 430060 Hubei China; 2 Department of Urology, Renmin Hospital of Wuhan University, No. 238 Jiefang Road, Wuchang District, Wuhan, 430060 Hubei China; 3 Department of Breast Surgery, The Affiliated Hospital of Guizhou Medical University, No. 28 Guiyi Road, Yunyan District, Guiyang, 550001 Guizhou China

**Correction: Experimental Hematology & Oncology (2023) 12:3** 10.1186/s40164-022-00363-1

In the Funding secion of this article [1], the grant number relating to National Natural Science Foundation of China was given incorrectly as No.8210114078 and should have been No.82103671.

**Funding**

This work was funded by the National Natural Science Foundation of China (Grant No.82103671).

The original article has been corrected.

## References

[1] Guo L, Kong D, Liu J, Zhan L, Luo L, Zheng W, Zheng Q, Chen C, Sun S (2023). Breast cancer heterogeneity and its implication in personalized precision therapy. Exp Hematol Oncol.

